# Edible Cannabis Legalization and Cannabis Poisonings in Older Adults

**DOI:** 10.1001/jamainternmed.2024.1331

**Published:** 2024-05-20

**Authors:** Nathan M. Stall, Shengli Shi, Kamil Malikov, Sping Wang, Paula A. Rochon, Michael P. Hillmer, Jonathan S. Zipursky

**Affiliations:** 1 Division of General Internal Medicine and Geriatrics, Sinai Health and the University Health Network, Toronto, Ontario, Canada; 2 Women’s Age Lab and Women’s College Research Institute, Women’s College Hospital, Toronto, Ontario, Canada; 3 Department of Medicine, University of Toronto, Ontario, Canada; 4 Digital and Analytics Strategy Division, Ontario Ministry of Health, Toronto, Canada; 5 Institute of Health Policy, Management and Evaluation, University of Toronto, Ontario, Canada; 6 Sunnybrook Research Institute, Toronto, Ontario, Canada; 7 Department of Medicine, Sunnybrook Health Sciences Centre, Toronto, Ontario, Canada

## Abstract

This cross-sectional study examines the association between edible cannabis legalization and emergency department visits for cannabis poisonings in older adults.

In October 2018, Canada legalized the sale of dried cannabis flowers for nonmedical use, and in January 2020, edible cannabis became legally available for retail.^1^ In California, legalization of all forms of nonmedical cannabis has been associated with increased cannabis-related emergencies in older adults (aged ≥65 years).^2^ Limited information exists on the specific health outcomes of nonmedical edible cannabis use in older adults^3^; thus, we examined the association between edible cannabis legalization and emergency department (ED) visits for cannabis poisoning in older adults residing in Ontario, Canada.

## Methods

This retrospective, population-based, cross-sectional study was approved by the Sunnybrook Health Sciences Centre research ethics board, which waived the need for informed consent because all data were deidentified. This study followed the STROBE reporting guideline.

We used linked Ontario Ministry of Health administrative data to examine ED visit rates for cannabis poisoning in older adults during 3 policy periods: prelegalization (January 2015 to September 2018); legalization period 1, which permitted the sale of dried cannabis flowers only (October 2018 to December 2019); and legalization period 2, which also permitted the sale of edible cannabis (January 2020 to December 2022). We identified ED visits where cannabis poisoning was the main or contributing reason (eAppendix in Supplement 1) and calculated rates per 100 000 person-years for older adults. We calculated incidence rate ratios (IRRs) using a Poisson regression model and 3-level categorical variable for each policy period. We adjusted models for age, sex, rurality, neighborhood income quintile, alcohol intoxication, cancer diagnosis, and dementia diagnosis (eAppendix in the Supplement). Data on race and ethnicity were unavailable. A 2-tailed type I error rate of .05 was the threshold for statistical significance. Statistical analyses were conducted using SAS, version 9.4 (SAS Institute, Inc).

## Results

During the 8-year study period, there were 2322 ED visits for cannabis poisoning in older adults (1041 women [44.8%]; 1281 men [55.2%]; median [IQR] age, 69.5 [67.3-73.8] years). Among patients with cannabis poisoning, 385 (16.6%) had concomitant alcohol intoxication, 895 (38.5%) cancer, and 151 (6.5%) dementia.

During legalization period 1, the rate of ED visits was substantially higher than prelegalization (15.4 vs 5.8 per 100 000 person-years; adjusted IRR, 2.00; 95% CI, 1.29-3.10) (Figure; Table). During legalization period 2, the rate of ED visits (21.1 per 100 000 person-years) was significantly greater than prelegalization (adjusted IRR, 3.08; 95% CI, 2.04-4.65).

**Figure.**
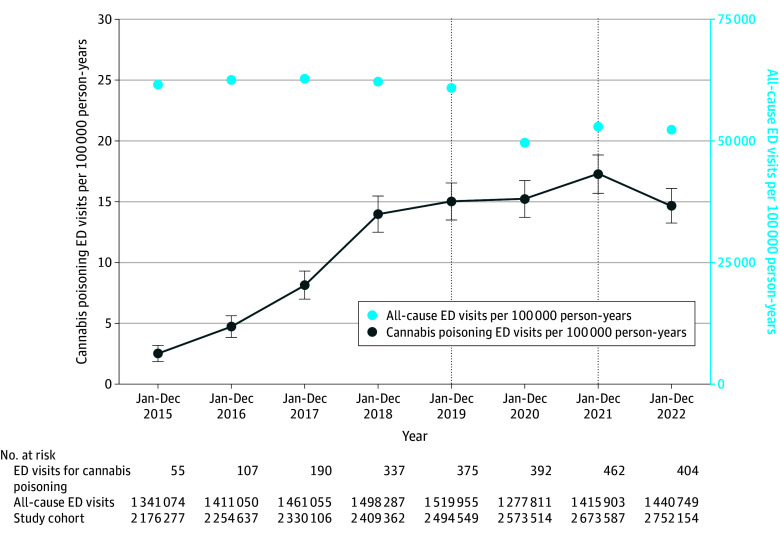
Annual Rates of Emergency Department (ED) Visits for Cannabis Poisoning in Ontario Older Adults, 2015-2022 The vertical dotted lines indicate the legalization of dried cannabis flower in October 2018 and legalization of edible cannabis in January 2020. Whiskers indicate 95% CIs.

**Table.** Multivariable Poisson Analysis for Factors Associated With Emergency Department Visits for Cannabis Poisoning in Ontario Older Adults, 2015-2022

Variable	Adjusted IRR (95% CI)^a^
Sociodemographic and clinical characteristics	
Aged 65-74 vs ≥75 y	2.21 (1.62-3.02)
Male sex	1.53 (1.14-2.06)
Urban vs rural residence	1.03 (0.77-1.38)
Neighborhood income quintile 1-3 (lower) vs 4-5 (higher)	0.93 (0.70-1.25)
Alcohol intoxication	4.20 (2.91-6.07)
Cancer diagnosis	0.93 (0.69-1.25)
Dementia diagnosis	0.71 (0.53-0.95)
Legalization period	
Legalization period 1 vs prelegalization	2.00 (1.29-3.10)
Legalization period 2 vs prelegalization	3.08 (2.04-4.65)
Legalization period 2 vs period 1	1.54 (1.11-2.13)

Abbreviation: IRR, incidence rate ratio.

^a^ Adjusted for age, sex, rurality, neighborhood income quintile, alcohol intoxication, cancer diagnosis, and dementia diagnosis (eAppendix in Supplement 1). We selected model covariates a priori based on a literature review and expert opinions of geriatric medicine, internal medicine, and pharmacology and toxicology specialists.^2^ We offset Poisson models by the log-transformed person-years at risk. A 2-tailed type I error rate of .05 was the threshold for statistical significance.

## Discussion

In this study, cannabis legalization in Canada was associated with increased rates of ED visits for cannabis poisoning in older adults. The largest increases occurred after edible cannabis became legally available for retail sale, a phenomenon similarly observed in Canadian children.^4^ Possible explanations include increases in accidental ingestion; ease of access; lack of age-specific dosing instructions; and absence of safe and effective treatment options for chronic pain, sleep disturbances, and behavioral and psychological symptoms of dementia. Older adults are at particularly high risk of adverse effects from cannabis due to age-related physiological changes, polypharmacy, drug interactions, and multimorbidity.^2^

Our study is limited to emergency department visit data and may underestimate the true magnitude of cannabis poisonings. Older adults may have sought care elsewhere or not at all, especially since the legalization of edible cannabis immediately preceded the COVID-19 pandemic. From the cross-sectional nature of our data, we cannot determine whether increased poisonings were directly attributable to edible cannabis or to broader commercialization of nonmedical cannabis.^5^ Furthermore, our findings may be influenced by other temporal trends and confounding by concurrent events, including the COVID-19 pandemic.

Our findings align with national US data showing that edible cannabis accounts for an increasing proportion of cannabis poisoning in older adults.^6^ Overall, this study shows the health outcomes of cannabis legalization and commercialization for older adults and highlights the consequences associated with edible cannabis. Jurisdictions with legalized cannabis should consider measures to mitigate unintentional exposure in older adults and age-specific dosing guidance.

## Notes

Supplement 1. **eAppendix.** Additional Methodological Details

Supplement 2.
Data Sharing Statement

## References

[1] Bill C-45: the cannabis act. Parliament of Canada. Accessed May 11, 2023. https://www.parl.ca/DocumentViewer/en/42-1/bill/C-45/royal-assent

[2] Han BH, Brennan JJ, Orozco MA, Moore AA, Castillo EM. Trends in emergency department visits associated with cannabis use among older adults in California, 2005-2019. J Am Geriatr Soc. 2023;71(4):1267-1274. doi:10.1111/jgs.18180 36622838 PMC10089945

[3] Wolfe D, Corace K, Butler C, . Impacts of medical and non-medical cannabis on the health of older adults: Findings from a scoping review of the literature. PLoS One. 2023;18(2):e0281826. doi:10.1371/journal.pone.0281826 36800328 PMC9937508

[4] Myran DT, Tanuseputro P, Auger N, Konikoff L, Talarico R, Finkelstein Y. Edible cannabis legalization and unintentional poisonings in children. N Engl J Med. 2022;387(8):757-759. doi:10.1056/NEJMc2207661 36001718

[5] Myran DT, Gaudreault A, Konikoff L, Talarico R, Liccardo Pacula R. Changes in cannabis-attributable hospitalizations following nonmedical cannabis legalization in Canada. JAMA Netw Open. 2023;6(10):e2336113. doi:10.1001/jamanetworkopen.2023.36113 37796504 PMC10556968

[6] Choi NG, Marti CN, DiNitto DM, Baker SD. Cannabis and synthetic cannabinoid poison control center cases among adults aged 50+, 2009-2019. Clin Toxicol (Phila). 2021;59(4):334-342. doi:10.1080/15563650.2020.1806296 32840426

