# Supplementary material for: Edible Cannabis Legalization and Cannabis Poisonings in Older Adults
Source: JAMA Intern Med. 2024 May 20;184(7):840–2. doi: 10.1001/jamainternmed.2024.1331 (PMC11106709; doi:10.1001/jamainternmed.2024.1331)
Supplement: Supplement 1. — eAppendix. Additional Methodological Details [file jamainternmed-e241331-s001.pdf]

Supplemental Online Content

Stall NM, Shi S, Malikov K, et al. Edible cannabis legalization and cannabis poisonings in older adults. *JAMA Intern Med*. Published online May 20, 2024. doi:10.1001/jamainternmed.2024.1331

eAppendix 1. Additional Methodological Details

This supplemental material has been provided by the authors to give readers additional information about their work.

© 2024 Stall NM et al. *JAMA Internal Medicine.*

eAppendix 1: Additional Methodological Details

1. **Ethics approval:** The study was approved by the research ethics board at Sunnybrook Health Sciences Centre.

2. **Emergency department visits for cannabis poisoning** were identified if the following International Classification of Diseases, 10th revision (ICD-10) codes were listed: T40.7 (poisonings by cannabis, including derivatives) or F12.X (mental and behavioral disorders due to use of cannabinoids).

3. **Patients with alcohol intoxication** were identified using diagnostic codes for alcohol-related emergencies in any of the following databases: Discharge Abstract Database (DAD), National Ambulatory Care Reporting System (NACRS), and the Ontario Mental Health Reporting System (OMHRS). The following International Classification of Diseases, 10th revision (ICD-10) codes were used: F100 to F109 (psychiatric); K700 to K704, K292, K709, K852, and K860 (gastrointestinal); R780, T510, T519, X45, X65, and Y15 (intoxication); and E244, G312, G621, G721, I426, P043, Q860, Q99304, and Q99305 (miscellaneous).

4. **Patients with cancer** were identified with the following values in population groupers held by the Ontario Ministries of Health and Long-Term Care: R01A, R02C, R03D, R04F, R06H, R07K, R08I, R09I, R10J, R11K, R12K, R13L, R14L, R15L, R17, and R18.

   References:

   i. Weir S, Steffler M, Li Y, Shaikh S, Wright JG, Kantarevic J. Use of the Population Grouping Methodology of the Canadian Institute for Health Information to predict high-cost health system users in Ontario. CMAJ. Aug 10 2020;192(32):E907-E912. doi:10.1503/cmaj.191297

   ii. Canadian Institute of Health Information. CIHI's Population Grouping Methodology 1.4, Overview and Outputs, 2023. Updated 2023. Accessed September 29, 2023. https://www.cihi.ca/sites/default/files/document/cihi-population-grouping-methodology-v1.4-overview-outputs-manual-en.pdf

5. **Patients with dementia** were identified by using validated administrative algorithm (1 hospitalization, 3 physician claims, or 1 drug, which was earliest):

   – one hospitalization billing with any diagnosis of dementia
   or
   – three physician claims in two years separated by at least 30 days with a diagnosis of dementia
   or
   – one prescription filled for an Alzheimer's Disease Related Dementias medication (cholinesterase inhibitor: *donepezil, galantamine, or rivastigmine*)

© 2024 Stall NM et al. *JAMA Internal Medicine.*

This algorithm has a sensitivity of 79.3% (confidence interval (CI) 72.9–85.8%), specificity of 99.1% (CI 98.8–99.4%), positive predictive value of 80.4% (CI 74.0–86.8%), and negative predictive value of 99.0% (CI 98.7–99.4%).

Reference:

i. Jaakkimainen RL, Bronskill SE, Tierney MC, et al. Identification of Physician-Diagnosed Alzheimer's Disease and Related Dementias in Population-Based Administrative Data: A Validation Study Using Family Physicians' Electronic Medical Records. *J Alzheimers Dis*. Aug 10 2016;54(1):337-49. doi:10.3233/JAD-160105

© 2024 Stall NM et al. *JAMA Internal Medicine.*
