# Supplementary material for: Edible Cannabis Legalization and Cannabis Poisonings in Older Adults
Source: JAMA Intern Med. 2024 May 20;184(7):840–2. doi: 10.1001/jamainternmed.2024.1331 (PMC11106709; doi:10.1001/jamainternmed.2024.1331)
Supplement: Supplement 2. — Data Sharing Statement [file jamainternmed-e241331-s002.pdf]

Data Sharing Statement

Stall. Edible Cannabis Legalization and Cannabis Poisonings in Older Adults. *JAMA Intern Med*. Published May 20, 2024. doi:10.1001/jamainternmed.2024.1331

Data
Data available: Yes
Data types: Deidentified participant data, Data dictionary
How to access data: kamil.malikov@ontario.ca
When available: With publication

Supporting Documents
Document types: Statistical/analytic code
How to access documents: kamil.malikov@ontario.ca
When available: With publication

Additional Information
Who can access the data: Researchers whose proposed use of the data has been approved.
Types of analyses: For a specified purpose.
Mechanisms of data availability: With a signed data access agreement.
Any additional restrictions: N/A.
